# A UAV image dataset for object detection with annotations generated using LabelImg and Roboflow

**DOI:** 10.1016/j.dib.2026.112483

**Published:** 2026-01-16

**Authors:** Anindita Das, Vinitha Hannah Subburaj, Yong Yang, Craig W. Bednarz

**Affiliations:** West Texas A&M University, Canyon, TX 79016, USA

**Keywords:** UAV, Image dataset, Object detection, YOLOv7, Annotation, Roboflow, LabelImg, Remote sensing, Computer vision

## Abstract

The dataset consists of drone images of cotton fields which were created to aid precision agriculture and machine learning-based weed detection research. The main goal is to enable the creation of object detection models for crop-weed differentiation while providing a standard for model evaluation. The dataset release serves two purposes: it supports the advancement of automated agricultural monitoring and sustainable farming practices, and it adds to the expanding research on AI solutions for agricultural productivity and environmental management.

Specifications Table

**Table:** 

Subject	Computer Sciences
Specific subject area	UAV Image Analysis, Object Detection
Type of data	Image files (JPG) and annotation files (YOLO format .txt)
How Data Were Acquired	UAV flights conducted over cotton fields in the Texas Panhandle using the Autel Robotics EVO II Dual 640T V3 drone equipped with a 50 MP 0.8″ RYYB CMOS sensor
Data Format	Raw (JPG images) and processed (YOLO-formatted .txt annotation files)
Parameters for Data Collection	UAV flight altitude (15 m), camera resolution (50 MP, 4096 × 3072 pixels), clear daytime lighting, and early-to-mid growth stage crop conditions.
Description of Data Collection	The drone collected images under clear weather conditions at high resolution across different cotton field conditions. The drone operated at a steady height to achieve consistent image resolution. The raw images underwent inspection for annotation purposes to label cotton crops and weeds which led to the development of an object detection model.
Data Source Location	Texas Panhandle, United States
Data Accessibility	The dataset is available in Mendeley Data at doi:10.17632/sx2tphzvcw.1 Direct URL: [https://data.mendeley.com/datasets/sx2tphzvcw/1]

## 1 Value of the data

• The dataset contains drone imagery at high resolution with labeled annotations of cotton crops and weeds to support precision agriculture model training and evaluation [1,2].
• The dataset offers value to researchers who focus on agricultural automation and weed detection and remote sensing for developing machine learning pipelines [1,3].
• The data allows researchers to reproduce and benchmark YOLOv7 deep learning models that detect crops from non-crop vegetation in actual field environments [6,7].
• The dataset enables sustainable agriculture through automated weed monitoring which decreases both manual labor requirements and herbicide usage.
• The dataset functions as an essential resource for agronomists together with AI developers and environmental scientists who want to enhance field-level decision-making and resource efficiency [2,7].

## 2 Background

Precision agriculture has experienced significant advancements through deep learning and computer vision applications which now perform weed detection and crop monitoring tasks. UAV-based imaging provides an efficient scalable method to obtain high-resolution real-time field data that causes minimal disruption to agricultural operations [11]. The detection performance of many models depends heavily on the quality and diversity of datasets they receive for training [1]. The majority of publicly accessible agricultural datasets face challenges because they either have insufficient annotation details or their geographic and seasonal coverage is restricted which hinders the development of robust and generalizable models [2,5]. Real field data for cotton cultivation datasets are difficult to find because they are not well represented.

This dataset was originally developed and used in our previously published study on weed detection in cotton fields using YOLOv7, which demonstrated high detection accuracy and practical applicability under field conditions [12].

The dataset presented gathers high-resolution UAV imagery of cotton fields in the Texas Panhandle during clear weather conditions during early to mid-growth stages. The YOLO format annotation system provides a structured resource for training and benchmarking object detection models in precision agriculture through each image. The dataset exists to help researchers and developers and agricultural professionals create data-driven solutions for field-level weed and crop monitoring through deep learning.

## 3 Data Description

The dataset consists of 366 high-resolution aerial images acquired by the Autel Robotics EVO II Dual

640T V3 drone over cotton fields in the Texas Panhandle, USA. Each image has a resolution of 4096 × 3072 pixels and was taken in clear weather conditions and consistent natural lighting during the early to mid-growth stages of cotton.

The dataset includes the following components:

• Image Files: Stored in JPG format, the images depict a variety of field conditions, including differences in crop density, weed distribution, and soil texture.
• Annotation Files: Stored in YOLO format (.txt), with one annotation file corresponding to each image. Each line in the annotation file includes a class ID (0 for weed, 1 for cotton) and bounding box coordinates normalized to the image dimensions.
• The annotations are provided as bounding boxes to support object detection tasks and do not include pixel-level segmentation masks.
• Classes:

○ Class 0: Weed

○ Class 1: Cotton

The predominant weed species present in the field included Palmer amaranth, Russian thistle, and kochia. These species were not annotated separately, as the dataset was designed for field-level weed detection rather than species-level classification; therefore, all weeds were grouped under a single class “0”. The LabelImg tool served to create all annotations manually. Roboflow performed additional preprocessing steps which included image resizing and format conversion to make the data compatible with YOLOv7 object detection framework [4].

Sample images in the dataset illustrate:

• Sparse and dense weed presence
• The cotton crops show well-defined row patterns
• The field shows different conditions due to irrigation, soil and plant development stages.

The dataset serves as a useful benchmark for precision agriculture model development and testing.

To provide transparency regarding dataset composition and enable assessment of class balance, the total number of annotated bounding boxes per class is summarized in Table 1.

**Table 1:** Distribution of annotated bounding boxes across classes.

Class	Label	Number of annotations
0	Weed	5713
1	Cotton	50,821
—	**Total**	**57,534**

This distribution reflects the dense spatial coverage of cotton plants relative to weeds observed in the field during the selected growth stages.

## 4 Experimental Design, Materials and Methods

### 4.1 UAV data acquisition

• Platform: A UAV (Autel Robotics EVO II Dual 640T V3) equipped with a high-resolution XL709 camera module was used for aerial imaging over cotton fields [8].
• Flight Parameters:

○ Altitude: Approximately 15 m above ground level.
○ Flight Speed**:** Approximately 3 m/s o Area Coverage: Rural agricultural fields located in the Texas Panhandle, USA.
○ Conditions: Images were captured under clear skies and optimal daylight, minimizing shadow interference and ensuring high visual clarity.

• Data Curation:

○ After the flight all images were checked for quality and images that were blurry, overexposed or redundant were excluded.
○ The selected images were standardized to 4096 × 3072 pixels and stored in JPG format with consistent metadata.

### 4.2 Annotation process

• Tools Used:

○ LabelImg: Utilized for manual annotation and generation of YOLO-compatible .txt files [9].
○ Roboflow: Used for preprocessing and format conversion to ensure compatibility with the YOLO object detection framework [10].

• Annotation Protocol:

○ Each image was manually annotated using bounding boxes to label two classes: weed (class 0) and cotton (class 1).
○ A standardized labeling scheme was used to ensure consistency across all images.
○ A subset of the dataset was processed through both LabelImg and Roboflow to validate consistency and format compatibility.

• Quality Assurance:

○ Annotations were cross‑verified to ensure class correctness, bounding box alignment, and adherence to the YOLO format. Cross‑verification is a quality‑control step used during manual annotation to reduce human error and improve label consistency by re‑examining a subset of annotated images after the initial labeling process [13].
  In this study, approximately 10 % of the annotated images were randomly selected for re‑review. These images were checked for labeling consistency and bounding box accuracy, and any discrepancies or inconsistencies identified during this process were manually corrected before finalizing the dataset.

### 4.3 Data post-processing

• After annotation, the images and label files were checked for correct formatting, file integrity, and consistency between image-label pairs. The finalized files were then organized in a clear YOLO-format structure so they are ready to be directly used for model training and further research.

## Limitations

The dataset contains UAV imagery that only includes cotton fields in the Texas Panhandle which limits its usefulness for other geographic areas with different environmental and soil and crop management conditions. The dataset contains images from early-to-mid growth stages only and shows consistent clear weather and lighting conditions which limits its ability to capture variations from different growth phases and cloud cover and low-light conditions. The manual annotation process included verification steps but small errors might persist because of human interpretation during the labeling process. The dataset provides bounding box annotations for object detection and does not include pixel-level segmentation masks, which may limit its direct applicability to semantic or instance segmentation tasks.

## Ethics Statement

This dataset does not involve any research experiment on humans or animals nor collect any data from online sources and social media platforms.

## Credit Author Statement

**Anindita Das**: Conceptualization, Methodology, Software, Data curation, Formal analysis, Writing – original draft. **Vinitha Hannah Subburaj**: Supervision, Project administration, Writing – review & editing, Corresponding author. **Yong Yang**: Supervision, Writing – review & editing. **Craig Bednarz**: Resources, Data provision, Field support.

## Data Availability

Mendeley DataA UAV Image Dataset for Object Detection with Annotations Generated Using LabelImg and Roboflow (Original data).
